# Sirolimus may improve bile excretion in *ABCB11* mutants: A case report of a patient with bile salt export pump deficiency

**DOI:** 10.1016/j.gendis.2025.101532

**Published:** 2025-01-15

**Authors:** Tao Zeng, Jiahui Pang, Yutian Chong, Guofang Tang, Yingying Liao, Xianghao Cai, Xiaolong Xiao, Yibo Zhang, Shuru Chen, Xinhua Li

**Affiliations:** a Department of Infectious Diseases, Key Laboratory of Liver Disease of Guangdong Province, The Third Affiliated Hospital of Sun Yat-Sen University, Guangzhou, Guangdong 510630, China; b Institute of Infectious Diseases, Guangzhou Eighth People's Hospital, Guangzhou Medical University, Guangzhou, Guangdong 510440, China

Progressive familial intrahepatic cholestasis type 2 (PFIC2), also known as bile salt export pump (BSEP) deficiency disease, is a rare autosomal recessive inherited liver disease caused by mutations in the *ABCB11* gene (located on chromosome 2q24-31), leading to impaired bile secretion.^1^ Over 200 distinct mutations in the *ABCB11* gene have been identified, including missense, nonsense, insertion, deletion, and splice site mutations.^1^ Compared with other types of PFIC, patients with BSEP deficiency are at a higher risk of progressing to cirrhosis and liver failure. Current treatment options for PFIC2 remain limited and frustrating. Liver transplantation, the only effective intervention, remains the sole definitive treatment for PFIC2. Nevertheless, its application is severely limited by the prohibitive cost and the scarcity of suitable liver donors. Addressing the pathogenesis of PFIC2 poses a significant clinical challenge. However, a recent study has shown that sirolimus may partially restore the bile excretion ability of *ABCB11* mutants in *abcb11* knockout Zebrafish models.^2^ This case report describes a patient with BSEP deficiency disease who responded favorably to sirolimus treatment.

A 15-year-old male presented with an 8-year history of recurrent jaundice, pruritus, fatigue, and dark urine. Physical examination demonstrated jaundice affecting the skin, mucous membranes, and sclera. Despite multiple visits to local hospitals seeking medical assistance, his symptoms remained unresolved. The liver ultrasound demonstrated uniform and dense echogenicity within the liver parenchyma, accompanied by hepatomegaly, porta hepatis lymphadenopathy, and splenomegaly. Histopathological analysis of the liver biopsy revealed chronic inflammatory alterations (G1S2) characterized by dilated bile ducts in the portal tract, and fibrous tissue proliferation with focal lymphocytic infiltration. Following an inconclusive initial liver biopsy, subsequent genetic analysis revealed compound heterozygous variants in the *ABCB11* gene (Fig. 1B, C and Table S1), confirming a diagnosis of PFIC2. Despite this clearer understanding of the underlying condition, the patient continued to experience recurrent symptoms, albeit with transient relief from local therapies. As the disease advanced, his clinical status deteriorated, necessitating referral to our hospital for more comprehensive management.

**Figure 1:**
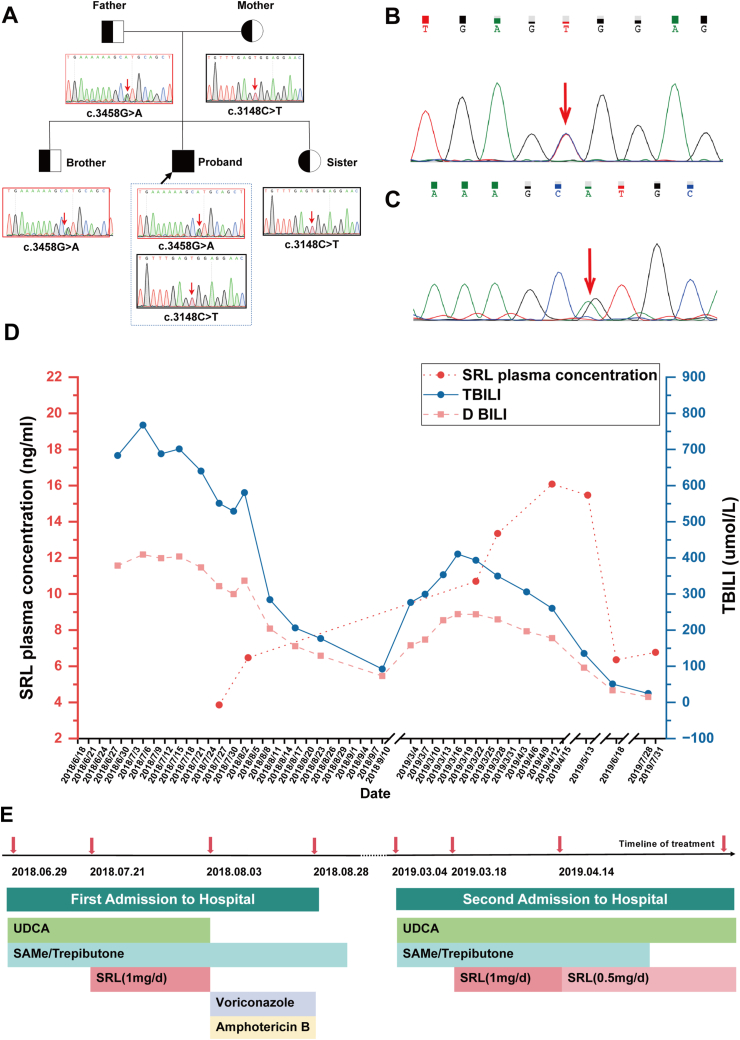
The comprehensive exome sequencing profile and the patient's clinical response to sirolimus therapy. **(A)** The variants were also identiﬁed in the patient's family members. **(B)** c.3148C > T (a mutation from cytosine to thymine at coding region nucleotide position 3148), leading to an amino acid change p.R1050C (from arginine to cysteine at amino acid position 1050), is also a missense mutation. **(C)** c.3458G > A (a mutation from guanine to adenine at coding region nucleotide position 3458), leading to an amino acid change p.R1153H (from arginine to histidine at amino acid position 1153), is a missense mutation. **(D)** Serum bilirubin trends. **(E)** Timeline of treatment of this patient. UDCA, ursodeoxycholic acid; SRL, Sirolimus; DBIL: direct bilirubin; SAMe: S-adenosylmethionine.

Following the confirmation of the diagnosis, we commenced conventional pharmacotherapy, incorporating ursodeoxycholic acid (UDCA), ademetionine, and trepibutone to augment bile excretion. However, the patient's condition progressively deteriorated, accompanied by an increasing bilirubin level. After an extensive review of the pertinent literature, our focus shifted decisively towards sirolimus, given its demonstrated efficacy in restoring bile acid excretion in *abcb11* knockout Zebrafish models.^2^ Following the initiation of sirolimus at a daily dose of 1 mg, we closely monitored the serum concentration, which stabilized at 6.47 ng/mL. Remarkably, after 14 days of treatment, there was a substantial reduction in total bilirubin (TBIL) from 701.3 μmol/L to 176.7 μmol/L, as well as a decline in direct bilirubin (DBIL) from 403.6 μmol/L to 129 μmol/L (Fig. 1D). These findings suggest a favorable response to sirolimus therapy. Regrettably, sirolimus treatment necessitated discontinuation due to a pulmonary fungal infection. Subsequently, the patient received voriconazole and amphotericin B antifungal therapy (Fig. 1E). Following a 25-day course of antifungal treatment, the patient's condition exhibited gradual improvement, leading to discharge with TBIL levels of 176.7 μmol/L.

However, six months following the initial hospitalization, the patient was readmitted due to complaints of yellow urine, generalized yellow discoloration of the body, and pruritus. Liver function tests indicated elevated levels of aspartate aminotransferase (AST, 109 U/L), alanine aminotransferase (ALT, 105 U/L), TBIL (276.5 μmol/L), and DBIL (158 μmol/L). Despite receiving standard medical therapy, the patient's bilirubin levels continued to rise, and the jaundice persisted. Drawing from our prior experience, we reintroduced sirolimus therapy, starting with an initial dose of 4 mg, followed by 1 mg daily. After four weeks, we adjusted the dosage to 0.5 mg daily for long-term maintenance, guided by a blood concentration of 16.08 mg/L. Subsequently, the patient exhibited improvement in yellow urine and skin discoloration, along with alleviation of itching. Notably, bilirubin levels progressively decreased (Fig. 1A). Consequently, the patient was discharged and continued regular sirolimus administration. Post-discharge, we maintained continuous monitoring of bilirubin and sirolimus blood levels. Over a follow-up period of more than 5 months, sirolimus effectively managed bilirubin levels and improved clinical symptoms.

The BSEP, encoded by the *ABCB11* gene, is the sole known transporter on the human liver canalicular membrane responsible for the translocation of bile salts into the canalicular lumen.^3^ The characteristic clinical features of PFIC2 encompass jaundice with yellowing of the skin and sclera, intractable pruritus, disturbances in lipid metabolism, fatigue, and steatorrhea (Table S2). PFIC2 is particularly aggressive, with a high likelihood of early liver failure and potential progression to hepatic malignancy.^4^ In this case, the proband harbors compound heterozygous missense mutations in the *ABCB11* gene, namely c.3458G > A (p.R1153H) and c.3148C > T (p.R1050C), inherited paternally and maternally, respectively (Fig. 1A). The proband's siblings, a brother and a sister, each carry one of these mutations (Fig. 1A). Both mutations are exceedingly rare in the general population and are not recognized as polymorphic loci. These genetic alterations have been previously associated with PFIC2.^5^ PFIC2 can be easily overlooked by clinicians due to its non-specific clinical presentation. Without early medical intervention, PFIC2 can lead to early liver cirrhosis and liver failure, resulting in serious consequences for patients. Drug therapy is typically the first-line treatment for PFIC2, with commonly used medications including UDCA, rifampicin, cholestyramine, and phenobarbital. However, most pharmacologic treatments have shown limited efficacy in patients with severe BSEP deficiency, who may ultimately require liver transplantation. Many patients undergo liver transplantation due to severe pruritus even before reaching end-stage liver disease. Consistent with previous studies, the patient in this case exhibited progressive jaundice and elevated bilirubin levels despite systematic treatment, leading to repeated hospitalizations. After reviewing the relevant literature and obtaining informed consent from the patient, sirolimus was considered as a treatment option.

Sirolimus, widely used as an immunosuppressant to prevent organ rejection post-transplantation, has recently been reported to offer additional benefits in the treatment of PFIC2, particularly in restoring bile excretion in patients with *ABCB11* mutations.^2^ In this case, systemic medical treatment failed to control a patient's increasing bilirubin levels and worsening condition. Sirolimus treatment was initiated as a last resort, and it produced surprising results. The patient's blood bilirubin levels were quickly controlled and gradually returned to normal, while symptoms such as itching and yellowing of the skin and sclera improved, leading to discharge from the hospital.

The exact mechanism by which sirolimus improves bilirubin excretion in patients with *ABCB11* mutations is not yet fully understood. As an mTOR inhibitor, sirolimus may restore bile excretion by recovering canalicular multidrug resistance protein 1 (MRP1) localization.^2^ This represents a promising avenue for further research and potential treatments for PFIC2 patients with *ABCB11* mutations. However, it is important to note that sirolimus is primarily metabolized by the liver P450 enzyme CYP3A4, which may cause liver damage, and regular monitoring of sirolimus blood concentration is necessary.

To the best of our knowledge, this is the first reported use of sirolimus for BSEP deficiency. Sirolimus may offer a potential treatment option for PFIC2 patients who do not respond to traditional therapies. However, there is currently insufficient clinical evidence supporting the use of sirolimus for this condition, and the precise mechanism by which it ameliorates cholestasis remains unclear and warrants further investigation. Nonetheless, additional research is required to establish the long-term safety and efficacy of sirolimus in the management of PFIC2.

## Ethics declaration

The studies involving human participant has been approved by the Ethics Committee of the Third Affiliated Hospital of Sun Yat-sen University (EY2022-247-01), and informed consent has been obtained from the patient.

## Funding

This study was supported by the 5010 Cultivation Program of Clinical Research of Sun Yat-Sen University (No. 2018024) and Guangzhou Science and Technology Plan Project (2023A03J0807).

## CRediT authorship contribution statement

**Tao Zeng:** Writing – original draft, Formal analysis. **Jiahui Pang:** Visualization, Data curation. **Yutian Chong:** Writing – original draft, Software, Data curation. **Guofang Tang:** Methodology, Funding acquisition. **Yingying Liao:** Writing – original draft, Data curation. **Xianghao Cai:** Investigation, Data curation. **Xiaolong Xiao:** Data curation. **Yibo Zhang:** Data curation. **Shuru Chen:** Investigation, Data curation. **Xinhua Li:** Data curation, Conceptualization.

## Conflict of interests

The authors declare that there are no competing interests.
