# Supplementary material for: Sirolimus may improve bile excretion in ABCB11 mutants: A case report of a patient with bile salt export pump deficiency
Source: Genes Dis. 2025 Jan 15;12(5):101532. doi: 10.1016/j.gendis.2025.101532 (PMC12166988; doi:10.1016/j.gendis.2025.101532)
Supplement: Multimedia component 1 [file mmc1.docx]

**Detailed Materials and Methods**

**Patient history**

**Medical Records from Other Hospitals**

A 15-year-old male from Guangdong Province, China, presented with an 8-year history of recurrent jaundice, pruritus, fatigue and yellow urination. The patient repeatedly sought medical assistance at local hospitals and received traditional Chinese medicine treatment, which provided temporary symptom relief lasting about six months before recurrence. As the disease progressed, his condition deteriorated despite treatment. In 2017, he was diagnosed with FIC2 at Meizhou People's Hospital after undergoing liver biopsy and genetic testing. The liver biopsy revealed chronic inflammatory changes (grade 1, stage 2) with dilated bile ducts in the portal area, fibrous tissue proliferation with focal lymphocyte infiltration, mild interface inflammation, hepatocyte swelling with widespread cholestasis, and few bile plugs in the small bile ducts. The initial liver biopsy did not lead to a definitive diagnosis. Subsequent genetic analysis identified compound heterozygous variants in the *ABCB11* gene, resulting in disease phenotypes of PFIC2. See Table S1 and Figure1 for details.

**Initial admission (May to August, 2018)**

Despite an increasingly clear diagnosis, the patient's disease condition worsened, prompting a referral to a higher-level medical institution for further treatment. On May 3rd, 2018, the patient was admitted to our hospital, with no history of viral hepatitis infection or genetic diseases in the family. Physical examination revealed jaundice of the skin, mucous membranes, and sclera throughout the body. The liver was enlarged, with a span of 6cm below the costal margin, exhibiting a soft consistency, smooth surface, and regular margins. The spleen was also enlarged, palpable 4cm below the costal margin, with no other notable findings. Upon admission, the following results were obtained: ALT: 99 U/L, AST: 86 U/L, TBIL: 660.5umol/L, DBIL: 527.1umol/L, γ-GGT: 25umol/L, ALP: 186U/L, TBA: 285.3umol/L.

Liver ultrasound examination revealed uniform and dense echogenicity of the liver parenchyma, with an enlarged liver (Left liver anteroposterior diameter: 87mm, left liver craniocaudal diameter: 112mm, left liver oblique diameter: 87mm, Right liver oblique diameter: 164mm) and mild enlargement of the porta hepatis lymph nodes. Chronic cholecystitis was evident, with multiple gallstones and thickened bile. The spleen was also enlarged (Splenic intercostal thickness: 51mm, Splenic longitudinal diameter: 176mm).

After confirming the diagnosis of FIC2, we prescribed drugs such as ursodeoxycholic acid (UDCA), ademetionine, and trepibutone to promote bile excretion. However, the patient's condition did not improve and their bilirubin levels remained high. Upon consulting numerous literatures, we turned our attention to sirolimus，which may be pharmacologically effective^7^. With the consent of the patient's family, we began administering sirolimus 1mg once daily from July 21, 2018 to promote bile excretion, and monitored the serum concentration at 6.47ng/ml. After 14 days of treatment, there was a progressive decrease in total bilirubin (TBil) (from 701.3 µmol/L to 176.7 µmol/L) and direct bilirubin (DBIL) (from 403.6 µmol/L to 129 µmol/L), indicating a positive response to sirolimus treatment. Unfortunately, sirolimus treatment had to be discontinued due to a pulmonary fungal infection, and the patient was treated with voriconazole and amphotericin B antifungal therapy. After a 25-day course of antifungal treatment, the patient's condition gradually improved, and they were discharged with bilirubin levels of 176.7umol/L and direct bilirubin levels of 129umol/L.

**Relapse after initial admission (March to April, 2019)**

However, six months after the initial hospitalization, the patient was readmitted to our hospital on March 4th, 2019, due to complaints of yellow urine, yellow discoloration of the body, and itching. Liver function tests revealed elevated levels of several enzymes, including AST (109U/L), ALT (105U/L), TBIL (276.5umol/L), DBIL (158umol/L), γ-GGT (44umol/L), and TBA (389.5umol/L). Despite receiving standard medical therapy, the patient's bilirubin levels increased, and the jaundice did not subside. Based on our experience from the last hospitalization, we decided to administer sirolimus treatment again, starting with an initial dose of 4mg, followed by 1mg per day on the second day. After four weeks, the dosage was adjusted to 0.5mg daily for long-term maintenance, based on a blood concentration of 16.08mg/L. Following this treatment, the patient's yellow urine and yellow skin staining improved, and the itching subsided. Furthermore, the bilirubin levels decreased progressively, as shown in Figure 1D. As a result, the patient was discharged from the hospital and continued to take sirolimus regularly. After admission, we monitored the liver function data and sirolimus blood concentration of the patients continuously for five months.

**Sequencing Methodology**

**DNA Extraction and Library Construction**

1. DNA Extraction: Extract 3-5 μg of DNA from blood samples and fragment it for amplification to construct a whole genome library containing the target genes.

2. Target Gene Capture: Use a liquid-phase capture kit developed by the company to isolate the target genes from the library.

3. Sequencing: Perform high-throughput sequencing using the Illumina HiSeq 2000 platform, ensuring an average depth of no less than 200X. Analyze the sequencing results.

**Capture Sequencing Workflow**

The patented liquid-phase capture kit is mixed with the client's whole genome library. The target region genes hybridize to probes. These probes, bound to magnetic beads via biotin-streptavidin interaction, allow the non-target DNA fragments to be washed away through elution. This process enriches the desired gene fragments, which are then sequenced using HiSeq 2000 for further analysis to identify relevant biological information.

**Library Preparation Steps**

3.1 Sample Library Preparation

1. Sonication:

- Use 3 μg of DNA diluted to 30 ng/μL with 1× low TE Buffer.

- Perform fragmentation with a Covaris S2 instrument using the following settings:

- Cycles: 3 × 60 s

- Water bath temperature: 4°C

- Duty cycle: 20%

- Intensity: 5

- Mode: Frequency sweeping

- Resulting fragment size: ~150 bp

2. End Repair:

- Add 75 μL fragmented DNA, 14 μL End Repair Reaction Buffer, and 11 μL End Repair Enzyme Mix (Mygenostics Inc.) to a total volume of 100 μL.

- Incubate at 20°C for 30 minutes.

- Purify the product using Beckman Ampure beads at a ratio of 1.8:1 (beads: reaction volume).

3. A-Tailing:

- Mix 32 μL of purified DNA, 15 μL A-Tailing Reaction Buffer, and 3 μL A-Tailing Enzyme Mix (Mygenostics Inc.) in a 50 μL reaction.

- Incubate at 37°C for 30 minutes.

- Purify the product using Beckman Ampure beads at a ratio of 1.8:1.

4. Adaptor Ligation:

- Mix 27 μL purified DNA, 40 μL Ligation Reaction Buffer, and 30 μL Ligation Enzyme Mix (Mygenostics Inc.) in a 70 μL reaction.

- Incubate at 25°C for 10 minutes.

- Purify the product using Beckman Ampure beads at a ratio of 1.8:1.

5. PCR Amplification:

- Reaction composition:

- H2O: 40 μL

- 5× Phusion HF buffer: 20 μL

- 10 mM dNTP: 2 μL

- DMSO: 5 μL

- Illumina PE primer #1 (100 μM): 1 μL

- Illumina PE primer #2 (100 μM): 1 μL

- Hotstart Phusion: 1 μL

- Template DNA: 30 μL

- Total volume: 100 μL

- PCR program:

- Stage 1: 98°C for 1 minute

- Stage 2: 9 cycles of (98°C for 20 seconds, 65°C for 30 seconds, 72°C for 30 seconds)

- Stage 3: 72°C for 5 minutes, then 4°C for 10 seconds

- For multiplexed samples, replace PE#2 with Illumina index sequences to distinguish sequencing data.

- Purify PCR products using Beckman Ampure beads at a ratio of 1.8:1.

3.2 Library Quality Assessment

1. Quantification:

- Use Nanodrop 2000 or Qubit to quantify 1 μL of the prepared library sample.

- Perform 1% agarose gel electrophoresis with 3 μL of the library sample. Expected fragment size: 300-500 bp.

2. Sample Integrity:

- Ensure a total library yield of at least 3 μg, with fragments appearing as a single band range rather than a discrete band to guarantee capture success.

- Short-term storage: 4°C; Long-term storage: -20°C.

Bioinformatics Analysis

4.1 SNP Analysis Workflow

1. Obtain raw short reads.

2. Remove sequencing adapters and low-quality data.

3. Align short reads to the human genome using SOAPaligner.

4. Analyze sequencing metrics, including read count, target region coverage, and average sequencing depth.

5. Identify genotypes in target regions using SOAPsnp.

6. Filter low-quality (quality score ≥20) and low-coverage (depth ≥10) SNPs.

7. Annotate SNPs using CCDS, NCBI 36.3, dbSNP (v130), and predict their impact on protein function using SIFT.

4.2 InDel Analysis Workflow

1. Align cleaned short reads to the human genome using Burrows-Wheeler Aligner (BWA).

2. Identify insertions and deletions (InDels) using GATK.

3. Annotate InDels using CCDS, NCBI 36.3, and dbSNP (v130), determining affected genes, coordinates, mRNA sites, coding sequence changes, and functional impact (e.g., amino acid insertions/deletions, frameshift mutations).

**Sirolimus Reagent Kit**

(Chemiluminescent Microparticle Immunoassay)

1×100 Tests/Box

Manual Preprocessing Procedure

Gently mix each sample (patient sample, calibrator, or control) by inverting it 5–10 times. For whole blood samples stored for extended periods, longer mixing is recommended. Visual inspection is advised to confirm thorough mixing.

After mixing, accurately pipette 150 µL of the sample into a microcentrifuge tube or an equivalent polypropylene centrifuge tube (e.g., round-bottom tubes). Use a separate centrifuge tube for each sample.

Add Pre-treatment Reagent:

a. Set the precision pipette to 300 µL. Using the pipette, accurately aspirate 300 µL of the ARCHITECT Sirolimus Pre-treatment Reagent from the yellow-labeled bottle.

b. Add 300 µL of the reagent into the centrifuge tube containing the sample. Ensure the pipette tip touches the wall of the centrifuge tube when adding the reagent.

Warning: Before adding the reagent to the next centrifuge tube, ensure the current tube is capped and vortex-mixed immediately.

c. After capping the tube, vortex vigorously for 5–10 seconds. Set the vortex mixer to the highest speed.

d. Place the capped tube in a 42°C heating block for 10 minutes, then centrifuge immediately.

Warning: Skipping the incubation step may lead to inaccurate test results.

Centrifuge the tubes:

Load the tubes into a microcentrifuge, ensuring rotor balance. Add balancing tubes if needed. The number of tubes in the centrifuge must be even.

Centrifuge at >9500×g RCF for at least 4 minutes or for 38,500 g-minutes.

After centrifugation, inspect the tubes:

Ensure the pellet is compact, and the supernatant is clear.

Prepare the sample for ARCHITECT i system testing:

Open the centrifuge tube cap and transfer the supernatant into a pre-treatment transfer tube.

Vortex the transfer tube for 5–10 seconds.

Load the transfer tube into the ARCHITECT sample rack.

Testing Procedure

Before first-time use, the microparticle reagent in the ARCHITECT Sirolimus Reagent Kit must be mixed to resuspend any particles settled during transport. After the initial loading, no further mixing is required.

Mixing the microparticle reagent:

Invert the bottle 30 times.

Visually inspect the reagent bottle to confirm particle resuspension. If particles remain adhered to the bottle, continue inverting until fully resuspended.

Once resuspended, cap the reagent bottle with a soft cap.

Install the ARCHITECT Sirolimus Reagent Kit onto the ARCHITECT i system for testing.

| **Table S1. DNA mutation information** | | | | | | | | |
| --- | --- | --- | --- | --- | --- | --- | --- | --- |
| Gene | Chromosome location | Exon | Nucleotide change | Amino acid change | Genotypes | Inheritance mode | Disease/phenotype | Mutation source |
| ABC  B11 | chr2-169783826 | exon2  6 | c.3458G  >A | p.R1153  H | Het | AR | 1. PFIC2  2. BRIC2 | Mother |
| ABC  B11 | chr2-  16978895 | exon2  4 | c.3148C  >T | p.R1050  C | Het | AR | 1. PFIC2  2. BRIC2 | Father |
| Het: Heterozygous, AR: autosomal recessive, PFIC2: progressive familial intrahepatic cholestasis Type 2, BRIC2: benign recurrent intrahepatic cholestasis Type 2. | | | | | | | | |

| **TableS2. Main Clinical Features of FIC2** |
| --- |
| ·Early onset, often within a few months after birth, especially in the neonatal period. |
| ·Rapid progress of the disease, with early onset of liver failure and liver, bile duct, and pancreatic cancer . |
| ·Mostly jaundice, mainly with elevated direct bilirubin. |
| ·Hepatosplenomegaly commonly present. |
| ·Intractable pruritus |
| ·Intrahepatic cholestasis with elevated total serum bile acids and usually normal or reduced γ-GGT |
| ·Liver function impairment. |
| ·Fat-soluble vitamin deficiency and steatorrhea |
| ·Growth failure. |
| ·Some patients respond to UDCA treatment. |
| - Liver transplantation is a common treatment option for many patients. |

FIC2：familial intrahepatic cholestasis type 2,γ-GGT: γ-Glutamyl Transpeptidase, UDCA, ursodeoxycholic acid.
